# Supplementary material for: Analysis of PPARγ Signaling Activity in Psoriasis
Source: Int J Mol Sci. 2021 Aug 10;22(16):8603. doi: 10.3390/ijms22168603 (PMC8395241; doi:10.3390/ijms22168603)
Supplement: Supplementary file 1 [file ijms-22-08603-s001.zip › Supplemental materials_Analysis of PPARg signaling activity in psoriasis/Pathway models/Models images and html files/Interleukin-17 and interleukin-22 signaling in psoriasis/8269.html]

PTPN11


# Protein PTPN11

|  |  |
| --- | --- |
| URN | urn:agi-llid:4903 |
| Total Entities | 0 |
| Connectivity | 3387 |
| Name | PTPN11 |
| Description | protein tyrosine phosphatase, non-receptor type 11 |
| Notes | The protein encoded by this gene is a member of the protein tyrosine phosphatase (PTP) family. PTPs are known to be signaling molecules that regulate a variety of cellular processes including cell growth, differentiation, mitotic cycle, and oncogenic transformation. This PTP contains two tandem Src homology-2 domains, which function as phospho-tyrosine binding domains and mediate the interaction of this PTP with its substrates. This PTP is widely expressed in most tissues and plays a regulatory role in various cell signaling events that are important for a diversity of cell functions, such as mitogenic activation, metabolic control, transcription regulation, and cell migration. Mutations in this gene are a cause of Noonan syndrome as well as acute myeloid leukemia. [provided by RefSeq, Aug 2016] |
| Primary Cell Localization | Cytoplasm |
| Class | Protein phosphatase |

---

|  |  |
| --- | --- |
| Pathway | Leptin and CCK8 Activate Nodose Ganglia Neuron |
|  | Natural Killer Cell Inhibitory Receptor Signaling |
|  | Peripheral T-Cell Tolerance |
|  | Do not Eat me\xc2\x9d Signal: Viable Cells Inhibits Phagocytosis |
|  | Eosinophil Survival by Cytokine Signaling |
|  | Mast-Cell Activation via IgE Signaling |
|  | Mast-Cell Activation without Degranulation |
|  | Mast-Cell Activation without Degranulation through KIT/F2R Signaling |
|  | Negative Acute Phase Proteins Synthesis |
|  | Positive Acute Phase Proteins Synthesis |
|  | IL6 Expression Targets -> Nociception |
|  | Proteins Involved in Atherosclerosis |
|  | Proteins Involved in Dilated Cardiomyopathy |
|  | IL22 Induces Keratinocyte Proliferation in Psoriasis |
|  | Insulin Resistance in Hepatocytes |
|  | Hepatic Stellate Cells in non-Alcoholic Fatty Liver Disease |
|  | Immune System Activation in Hyperthyroidism |
|  | Proteins Involved in Helicobacter Infections |
|  | CagA Phosphorylation Dependent Signaling |
|  | Proteins Involved in Acute Myeloid Leukemia |
|  | IL6/IGF1/VEGFA Signaling in Multiple Myeloma |
|  | Multiple Myeloma |
|  | Mast-Cell Activation in Asthma |
|  | Mast-Cell Related Chemokines Signaling in Asthma |
|  | IL6 Promotes Inflammation in Muscular Dystrophies |
|  | Leptin Role in Endometriosis |
|  | Noreadrenaline/Gq Expression Targets |
|  | Epinephrine/Gq Expression Targets |
|  | SELE -> ELK-SRF Signaling |
|  | Erythropoietin -> ELK/SRF Expression Targets |
|  | PECAM1 Expression Targets |
|  | GH1/GHR -> NF-kB/ELK/SRF/MYC Expression Targets |
|  | CSH1/GHR Expression Targets |
|  | Receptors and Adaptor Proteins Activated in Cancer |
|  | Proteins with Altered Expression in Cancer-Associated Sustaining of Proliferative Signaling |
|  | Genes with Mutations in Cancer-Associated Sustaining of Proliferative Signaling |
|  | Effector T-cell Inactivation in Cancer Immune Escape |
|  | PECAM -> SP1 Signaling |
|  | IL6R -> CEBP/ELK/SRF Signaling |
|  | IL6 Expression Targets |
|  | Leptin -> ELK/SRF Expression Targets |
|  | LPA Expression Targets |
|  | AGT -> TP53 Expression Targets |
|  | AGT -> CREB Expression Targets |
|  | AGT -> ELK/SRF Expression Targets |
|  | PRL/GHR -> NF/kB/ELK/SRF/MYC Expression Targets |
|  | AXL Receptor Tyrosine Kinase Signaling |
|  | ROS and RNS in Vasoconstriction and Vasodilation Regulation |
|  | ROS in Angiotensin Mediated Cardiovascular Remodeling and Hypertrophy |
|  | sialic |
|  | 2\_Interleukin-17 and interleukin-22 signaling in psoriasis |
|  | 2\_Defective tolerance of autoreactive T-cell in T1D |
|  | Interleukin-17 and interleukin-22 signaling in psoriasis |

---

|  |  |
| --- | --- |
| Group | Genes with Mutations Associated with Acute Myeloid Leukemia |
|  | Genes Associated with Syndromic Epilepsy |

---

|  |  |
| --- | --- |
| MedScan ID | 4903 |

---

|  |  |
| --- | --- |
| LocusLink ID | 4903 |
|  | 5781 |
|  | 19247 |
|  | 25622 |

---

|  |  |
| --- | --- |
| Alias | Src homology phosphatase-2 |
|  | phosphotyrosine phosphatase 1D |
|  | protein tyrosine phosphatase SH-PTP2 |
|  | RIKEN cDNA 2700084A17 gene |
|  | protein tyrosine phosphatase-2 |
|  | protein tyrosine phosphatase, nonreceptor type 11 isoform II |
|  | Src homology region phosphatase SHP2 |
|  | Src homology phosphatase SHP2 |
|  | protein tyrosine phosphatase, non receptor type 11 |
|  | PTNB\_HUMAN |
|  | phosphatase SHP2 |
|  | Noonan syndrome 1 gene |
|  | Src homology region 2 domain-containing phosphatase 2 |
|  | OTTMUSP00000017430 |
|  | Src homology region 2 domain-containing protein tyrosine phosphatase SHP-2 |
|  | protein-tyrosine phosphatase SYP |
|  | SH2 domain-containing protein tyrosine phosphatase-2 |
|  | protein tyrosine phosphatase, nonreceptor type 11 (Noonan syndrome 1) |
|  | protein tyrosine phosphatase, non receptor type 11 (Noonan syndrome 1) |
|  | SH2 domain-containing protein tyrosine phosphatase II |
|  | Noonan syndrome 1 protein |
|  | SH-PTP2 |
|  | BPTP3 |
|  | SHP2 |
|  | protein tyrosine phosphatase, nonreceptor type 11 isoform 1 |
|  | Src homology region phosphotyrosyl phosphatase SHP2 |
|  | nonreceptor protein tyrosine phosphatase 11 |
|  | protein tyrosine phosphatase, non receptor type 11 isoform 1 |
|  | Src homology region domain 2 containing protein phosphatase 2 |
|  | protein tyrosine phosphatase, non receptor type 11 isoform 2 |
|  | non receptor protein tyrosine phosphatase 11 |
|  | NS I |
|  | protein tyrosine phosphatase SHP2 |
|  | PTP1D |
|  | tyrosine phosphatase 2C |
|  | SHPTP2 |
|  | SHP2/pp75 |
|  | MGC14433 |
|  | protein tyrosine phosphatase, nonreceptor type 11 isoform I |
|  | Src homology phosphotyrosyl phosphatase SHP2 |
|  | Src homology 2 domain protein tyrosine phosphatase SHP-2 |
|  | SH-PTP3 |
|  | protein tyrosine phosphatase, non receptor type 11 isoform II |
|  | Src homology 2 domain-containing protein tyrosine phosphatase SHP-2 |
|  | METCDS |
|  | SHP2 phosphatase |
|  | SH-PTP2 protein tyrosine phosphatase nonreceptor type 11 |
|  | 2700084A17 |
|  | Noonan syndrome I gene |
|  | protein tyrosine phosphatase, nonreceptor type 11 isoform 2 |
|  | protein-tyrosine phosphatase 1D |
|  | AW536184 |
|  | Src homology region phosphatase-2 |
|  | PTP2C |
|  | PRO1847 |
|  | Src homology domain 2 containing protein phosphatase 2 |
|  | SH2 domain-containing protein tyrosine phosphatase SHP-2 |
|  | PTNB |
|  | PTPN11 |
|  | 2700084A17Rik |
|  | OTTHUMP00000166108 |
|  | Noonan syndrome I protein |
|  | protein-tyrosine-phosphatase, nonreceptor type 11 |
|  | Src homology region 2 domain protein tyrosine phosphatase SHP-2 |
|  | protein tyrosine phosphatase, non receptor type 11 isoform I |
|  | SH-PTP2 protein tyrosine phosphatase non receptor type 11 |
|  | CFC |
|  | NS1 |
|  | JMML |
|  | PTP-1D |
|  | PTP-2C |
|  | protein-tyrosine phosphatase 2C |
|  | tyrosine-protein phosphatase non-receptor type 11 |
|  | Syp |
|  | SAP-2 |
|  | SHP-2 |
|  | SH-PTP2 protein tyrosine phosphatase, non-receptor type 11 |
|  | encodes catalytic domain |
|  | SH-PTP2 protein tyrosine phosphatase non-receptor type 11 |
|  | null |
|  | protein tyrosine phosphatase, non-receptor type 11 (Noonan syndrome 1) |
|  | protein tyrosine phosphatase, non-receptor type 11 isoform I |
|  | protein tyrosine phosphatase, non-receptor type 11 isoform 2 |
|  | protein tyrosine phosphatase, non-receptor type 11 |
|  | protein tyrosine phosphatase, non-receptor type 11 isoform 1 |
|  | protein tyrosine phosphatase, non-receptor type 11 isoform II |

---

|  |  |
| --- | --- |
| EC Number | 3.1.3.48 |

---

|  |  |
| --- | --- |
| GO ID | 0031748 |
|  | 0005070 |
|  | 0050839 |
|  | 0005158 |
|  | 0043560 |
|  | 0004726 |
|  | 0051428 |
|  | 0043274 |
|  | 0004721 |
|  | 0001784 |
|  | 0019904 |
|  | 0019901 |
|  | 0004725 |
|  | 0030971 |
|  | 0060020 |
|  | 0000077 |
|  | 0038127 |
|  | 0031295 |
|  | 0033277 |
|  | 0000187 |
|  | 0036302 |
|  | 0007411 |
|  | 0007420 |
|  | 0071345 |
|  | 0071364 |
|  | 0071260 |
|  | 0021697 |
|  | 0019221 |
|  | 0048013 |
|  | 0007173 |
|  | 0060325 |
|  | 0008543 |
|  | 0048806 |
|  | 0042593 |
|  | 0007507 |
|  | 0048873 |
|  | 0042445 |
|  | 0009755 |
|  | 0048839 |
|  | 0007229 |
|  | 0070102 |
|  | 0061582 |
|  | 0050900 |
|  | 0035855 |
|  | 0032528 |
|  | 0035264 |
|  | 0048609 |
|  | 0033629 |
|  | 0051463 |
|  | 0060125 |
|  | 0046676 |
|  | 0048011 |
|  | 0035265 |
|  | 0035335 |
|  | 0030168 |
|  | 0030220 |
|  | 0048008 |
|  | 0070374 |
|  | 0046326 |
|  | 0046887 |
|  | 0046628 |
|  | 0032728 |
|  | 0032755 |
|  | 0045931 |
|  | 0050731 |
|  | 0051897 |
|  | 0032760 |
|  | 0033628 |
|  | 0043254 |
|  | 0046825 |
|  | 0060338 |
|  | 0006641 |
|  | 0005737 |
|  | 0005829 |
|  | 0005739 |
|  | 0005654 |
|  | 0005634 |
|  | 0032991 |
|  | 0016787 |
|  | 0016791 |
|  | 1990782 |
|  | 0007409 |
|  | 0016311 |
|  | 0006629 |
|  | 0046888 |
|  | 0009967 |
|  | 0006470 |
|  | 0030054 |
|  | 0016020 |
|  | 0001725 |
|  | 0040014 |
|  | 0016303 |
|  | 0046934 |
|  | 0046854 |
|  | 0036092 |
|  | 0048015 |
|  | 2001275 |
|  | 0014066 |
|  | 0043234 |
|  | 0038095 |
|  | 0007596 |
|  | 0045087 |
|  | 0008286 |
|  | 0060333 |
|  | 0060334 |
|  | 0060337 |

---

|  |  |
| --- | --- |
| KEGG ID | hsa:5781 |
|  | mmu:19247 |
|  | rno:25622 |

---

|  |  |
| --- | --- |
| Organism | Homo sapiens {Organism urn:agi-taxid:9606} |
|  | Mus musculus {Organism urn:agi-taxid:10090} |
|  | Rattus norvegicus {Organism urn:agi-taxid:10116} |
|  | Homo sapiens |
|  | Mus musculus |
|  | Rattus norvegicus |

---

|  |  |
| --- | --- |
| Mouse chromosome position | 5 |

---

|  |  |
| --- | --- |
| OMIM ID | 176876 |
|  | 607785 |
|  | 151100 |
|  | 156250 |
|  | 163950 |

---

|  |  |
| --- | --- |
| Rat chromosome position | 12q16 |

---

|  |  |
| --- | --- |
| Hugo ID | 9644 |
|  | HGNC:9644 |

---

|  |  |
| --- | --- |
| Human chromosome position | 12q24.13 |
|  | 12q24 |

---

|  |  |
| --- | --- |
| Swiss-Prot Accession | Q06124 |
|  | Q06124.2 |
|  | P35235 |
|  | P35235.2 |
|  | P41499 |
|  | P41499.4 |
|  | A8K1D9 |
|  | Q96HD7 |
|  | Q3TQ84 |
|  | Q64509 |
|  | Q6PCL5 |
|  | Q62626 |

---

|  |  |
| --- | --- |
| PIR ID | JN0805 |
|  | A46209 |
|  | A53593 |

---

|  |  |
| --- | --- |
| GenBank ID | NC\_000012 |
|  | NM\_001330437 |
|  | NP\_001317366 |
|  | NM\_002834 |
|  | NP\_002825 |
|  | XM\_017019722 |
|  | XP\_016875211 |
|  | XM\_011538613 |
|  | XP\_011536915 |
|  | NM\_080601 |
|  | NP\_542168 |
|  | NG\_007459 |
|  | AC004086 |
|  | AC004216 |
|  | CH471054 |
|  | EAW98011 |
|  | EAW98012 |
|  | AK289854 |
|  | BAF82543 |
|  | AK312147 |
|  | BAG35082 |
|  | AU123593 |
|  | BC007869 |
|  | BC008692 |
|  | AAH08692 |
|  | BC025181 |
|  | AAH25181 |
|  | BC030949 |
|  | AAH30949 |
|  | BF515187 |
|  | BQ007989 |
|  | BT007106 |
|  | AAP35770 |
|  | BX537632 |
|  | CN258646 |
|  | D13540 |
|  | BAA02740 |
|  | DA498964 |
|  | DB450422 |
|  | EU779706 |
|  | ACE60554 |
|  | EU779707 |
|  | ACE60555 |
|  | EU779708 |
|  | ACE60556 |
|  | L03535 |
|  | AAA36611 |
|  | L07527 |
|  | AAA17022 |
|  | L08807 |
|  | S39383 |
|  | S78088 |
|  | AAB21148 |
|  | U79291 |
|  | X70766 |
|  | CAA50045 |
|  | Q06124 |
|  | NC\_000071 |
|  | NM\_001109992 |
|  | NP\_001103462 |
|  | NM\_011202 |
|  | NP\_035332 |
|  | AC110037 |
|  | AC127553 |
|  | CH466529 |
|  | EDL19739 |
|  | AK011468 |
|  | BAB27639 |
|  | AK012560 |
|  | AK135991 |
|  | AK147354 |
|  | AK159501 |
|  | BAE35134 |
|  | AK159587 |
|  | BAE35207 |
|  | AK163809 |
|  | BAE37500 |
|  | BC003980 |
|  | BC028513 |
|  | AAH28513 |
|  | BC057398 |
|  | AAH57398 |
|  | BC059278 |
|  | AAH59278 |
|  | BM899214 |
|  | CF751511 |
|  | D84372 |
|  | BAA12328 |
|  | S59187 |
|  | AAB26343 |
|  | P35235 |
|  | NC\_005111 |
|  | NM\_001177593 |
|  | NP\_001171064 |
|  | NM\_013088 |
|  | NP\_037220 |
|  | AC\_000080 |
|  | AABR07036375 |
|  | AABR07036376 |
|  | AAHX01072549 |
|  | AAHX01072550 |
|  | CH473973 |
|  | EDM13752 |
|  | D83016 |
|  | BAA74951 |
|  | U05963 |
|  | AAA19133 |
|  | U09307 |
|  | AAA20543 |
|  | U57499 |
|  | AAB02229 |
|  | P41499 |
|  | XM\_006719526 |
|  | XP\_006719589 |
|  | XM\_006719527 |
|  | XP\_006719590 |
|  | NC\_018923 |
|  | AMYH02027223 |
|  | AMYH02027224 |
|  | AC\_000027 |
|  | AAHY01048914 |
|  | AAHY01048915 |
|  | AAHY01048916 |
|  | AAHY01048917 |
|  | AAHY01048918 |
|  | AAHY01048919 |
|  | AC\_000144 |
|  | ABBA01019934 |
|  | ABBA01019935 |
|  | ABBA01019936 |
|  | ABBA01019937 |

---

|  |  |
| --- | --- |
| Swiss-Prot ID | PTN11\_HUMAN |
|  | PTN11\_MOUSE |
|  | PTN11\_RAT |

---

|  |  |
| --- | --- |
| Cell Localization | Cytoplasm |
|  | Nucleus |

---

|  |  |
| --- | --- |
| Ensembl ID | ENSG00000179295 |
|  | ENSP00000489597.1 |
|  | ENST00000635625.1 |
|  | ENSP00000340944.2 |
|  | ENST00000351677.6 |
|  | ENSP00000376376.1 |
|  | ENST00000392597.5 |
|  | ENSMUSG00000043733 |
|  | ENSMUSP00000098333.2 |
|  | ENSMUST00000100770.8 |
|  | ENSMUSP00000058757.7 |
|  | ENSMUST00000054547.8 |
|  | ENSRNOG00000030124 |
|  | ENSRNOP00000041842.5 |
|  | ENSRNOT00000046323.5 |
|  | ENSP00000340944 |
|  | ENST00000351677 |
|  | ENSP00000376376 |
|  | ENST00000392597 |
|  | ENSP00000489597 |
|  | ENST00000635625 |
|  | ENSMUSP00000098333 |
|  | ENSMUST00000100770 |
|  | ENSMUSP00000058757 |
|  | ENSMUST00000054547 |
|  | ENSRNOP00000041842 |
|  | ENSRNOT00000046323 |

---

|  |  |
| --- | --- |
| MGI ID | MGI:99511 |

---

|  |  |
| --- | --- |
| RGD ID | 3447 |

---

|  |  |
| --- | --- |
| Unigene ID | Hs.506852 |
|  | Mm.8681 |
|  | Rn.98209 |

---

|  |  |
| --- | --- |
| Homologene ID | 2122 |

---

|  |  |
| --- | --- |
| Shape | 2Triangles |

---
